# Hemophagocytic lymphohistiocytosis secondary to visceral leishmaniasis in an endemic area in the north of Minas Gerais, Brazil

**DOI:** 10.1590/0037-8682-0491-2019

**Published:** 2020-06-22

**Authors:** Fernando Henrique Guimarães de Carvalho, Jamille Fernandes Lula, Leandro de Freitas Teles, Antônio Prates Caldeira, Sílvio Fernando Guimarães de Carvalho

**Affiliations:** 1 Unimontes, Hospital Universitário Clemente de Faria, Montes Claros, MG, Brasil.; 2 Unimontes, Departamento de Saúde da Mulher e da Criança, Montes Claros, MG, Brasil.; 3 Fundação Hemominas, Montes Claros, MG, Brasil.

**Keywords:** Visceral Leishmaniasis, Hemophagocytic lymphohistiocytosis, Hemophagocytic syndrome

## Abstract

**INTRODUCTION:**

Visceral leishmaniasis (VL) is an ill-studied disease that is endemic to several regions of Brazil. It is often complicated by hemophagocytic lymphohistiocytosis (HLH), a potentially fatal disorder resulting from excessive non-malignant activation/proliferation of T lymphocytes and macrophages. Considering the overlapping clinical and laboratory characteristics of these diseases, diagnosing HLH is a challenge. Therefore, tracking the association between VL and HLH is necessary in endemic areas. Although HLH can be inapparent and resolve with antileishmanicides, this may not always occur. HLH causes high lethality; therefore, immunosuppressive therapy should be instituted immediately in order to avoid a fatal outcome.

**METHODS:**

We described the epidemiological, clinical, laboratory, and therapeutic profile of this association in a region of Brazil endemic for VL.

**RESULTS:**

We presented 39 patients with this association in a retrospective cohort of 258 children who were admitted from January 2012 to June 2017. Of the 39 patients, 31 were from urban areas (79.5%), and 21 (53%) were males. The mean age and weight were 2.86 (2.08) years and 14.03 (5.96) kg, respectively. The main symptoms were fever (100%), hepatosplenomegaly (100%), pallor of the skin and mucosa (82.5%), edema (38.5%), bleeding (25%), and jaundice (7.5%). Hemophagocytosis was identified in 16/37 (43.24%) patients, and direct examination revealed that 26/37 (70.27%) patients were positive for VL. The patients were treated as recommended by the Ministry of Health.

**CONCLUSIONS:**

It was observed that HLH is a common complication in endemic areas, and its diagnosis must consider the overlapping of clinical characteristics and pancytopenia.

## INTRODUCTION

Visceral leishmaniasis (VL) is a public health concern in developing countries. The overall annual incidence has been estimated at approximately 200,000 to 400,000 cases, with a mortality rate of 10% per year^1^. In Brazil, VL is a zoonosis caused by *Leishmania infantum*, with the domestic dog serving as the main reservoir and *Lutzomyia longipalpis* (sand fly) as the vector of greater epidemiological importance. More than 95% of the reported cases of VL in Latin America occur in Brazil, in more than 40% of children aged less than 10 years. The occurrence of VL is rapidly increasing, and concomitantly, there is an increase in its lethality, despite the currently available therapeutic options^2^. Patients with fever for a period ≥ 7 days, along with splenomegaly or hepatosplenomegaly, pallor of the skin and mucosa, and pancytopenia or peripheral bicytopenia are suspected of harboring the disease. 

**FIGURE 1:**
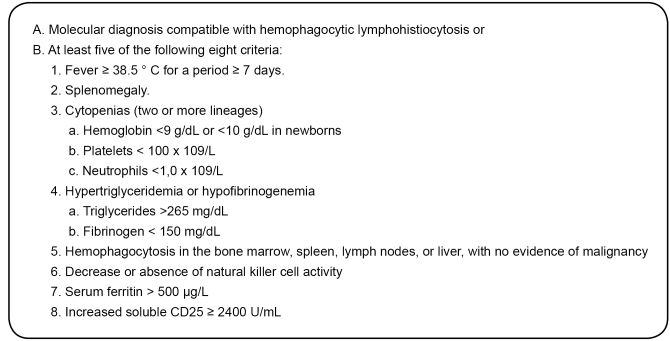
Criteria for the diagnosis of hemophagocytic lymphohistiocytosis. **Source:** Histiocyte Society-Treatment Protocol of The Second International HLH Study 2004.

Hemophagocytic lymphohistiocytosis (HLH), or hemophagocytic syndrome (HPS), poses a significant diagnostic and therapeutic challenge. According to the Histiocyte Society^3^ ^,^ ^4^, there are two types of HLH, namely, the primary or genetic form, also known as familial HLH, and a secondary or acquired form^5^ ^,^ ^6^, which occurs in patients with no family history or known genetic cause for the syndrome. Five of the eight criteria should be present to establish the diagnosis and initiate treatment (Figure 1).

HLH findings include persistent fever, hepatosplenomegaly, hypertriglyceridemia, hypofibrinogenemia, hyperferritinemia, cytopenia, liver dysfunction, decreased natural killer cell (NK) and IL-2 receptor (soluble r-CD25) activity, and the presence of hemophagocytosis in the bone marrow, spleen, and liver^5^ ^,^ ^6^ ^,^ ^7^. It is a potentially fatal disorder resulting from excessive non-malignant activation/proliferation of T lymphocytes and macrophages. Viral, bacterial, fungal, and parasitic infections, as well as autoimmune and neoplastic diseases, can trigger this disorder^4^. 

The literature considers VL/HLH association a rare occurrence. The first case of VL/HLH occurred in Greece in 1984, and by December 2012, forty-five pediatric cases had been described, with a mortality rate of 13.5%^8^. Given the scarcity of data in the literature, this retrospective study aimed at describing the clinical and laboratory presentations of VL, complicated with HLH, in children monitored at the Pediatrics Department of the University Hospital, Montes Claros State University (HU-UNIMONTES) between January 2012 and June 2017.

## METHODS

### Study design

This was a retrospective study of patients with an association between VL and HLH.

### Location, data source, and study period

The study was conducted at the Clemente de Faria University Hospital (HUCF) of the Montes Claros State University, which is the only reference hospital for the treatment of infectious and contagious diseases, including VL, in the entire northern region of Minas Gerais.

The data was collected from the medical records of children between the ages of zero and twelve years, who were admitted to the HUCF from January 2012 to June 2017 with a diagnosis of VL. 

### Ethical considerations

The study followed all ethical precepts and was conducted in accordance with the principles laid in resolution 466/12. The study protocol was approved by the Ethics Committee of the Montes Claros State University, Process No. 2050, and the institution authorized data collection. 

### Diagnostic tests

Patients who presented with the clinical disease (fever for a period ≥ 7 days, splenomegaly or hepatosplenomegaly, and pallor of the skin and mucosa) with pancytopenia or peripheral bicytopenia were suspected to have a diagnosis of VL. The diagnosis was confirmed with the confirmation of *Leishmania* in the smear of the bone marrow aspirate and/or a rapid positive test for VL (Kalazar Detect^®^, InBios, Seattle and IT Leish^®^, Bio-Rad, Marnes-la-Coquette). 

The presence of at least five of the six investigated criteria established by the Lymphohistiocytosis Hemophagocytic Study Group in 2004 was considered for the diagnosis of HLH^9^.

### Concept of cure

‘Cure’ was defined as complete remission of clinical signs and symptoms, associated with the normalization of changes in laboratory parameters observed at baseline, without evidence of recurrence in twelve months.

### Study variables

The demographic variables described were age, gender, and origin (rural or urban). The clinical variables were fever, hepatomegaly, splenomegaly, pallor, hemorrhage, jaundice, edema, nutritional status, presence of co-infections, time of disease onset, and discharge conditions. Laboratory variables were represented by blood count, platelet count, prothrombin activity, CRP (C-reactive protein), hemophagocytosis imaging research, direct examination of bone marrow aspirate and rapid tests for VL (Kalazar Detect^®^ and IT Leish^®^, and HIV (HIV Tri Line - K087^®^, Bioclin, Belo Horizonte). The levels of bilirubin, transaminases, amylase, lipase, urea, creatinine, triglycerides, ferritin, total proteins, and fractions were also estimated. The specific treatments used for VL were N-methylglucamine Antimony 20 mg.kg^-1^.day^-1^ for 20 consecutive days, Amphotericin B deoxycholate 1 mg.kg^-1^.day^-1^ for 14 consecutive days, and liposomal Amphotericin B 3 mg.kg^-1^.day^-1^ for 7 consecutive days or 4 mg.kg^-1^.day^-1^ for 5 consecutive days. Additional treatments, such as corticotherapy, antibiotic therapy, and blood components, were also identified. Image examinations were performed in the indicated cases. 

A standardized study questionnaire, developed by a group of researchers, was used to collect data from each patient.

### Statistical Analysis

Statistical analysis was performed using SPSS for Windows, version 18.0 (SPSS Inc., Chicago, IL, USA). 

## RESULTS

During the study period (January 2012 to June 2017) 258 children aged 9 years or younger, with a diagnosis of VL, were admitted to the Clemente de Faria University Hospital, Montes Claros State University-UNIMONTES. Among these 258 children, 39 (15.1%) met the diagnostic criteria of having an association between VL and HLH, and thirty-one out of 39 children with a VL/HLH association were from urban areas (79.5%). All patients lived in the north of the state of Minas Gerais, and the majority were from the city of Montes Claros. The mean age of the patients was 2.86 (2.08) years (ranging from five months old to nine years and two months old), with a median of two years and one month, and 90% of the patients were younger than five years old. Among 39 patients, 21 (53%) were males. The mean weight at admission was 14.03 (5.96) kg (ranging from 6.4 kg to 28 kg), with a median of 12 kg. Although no cases of malnutrition were identified, overweight cases were observed. 

The primary signs and symptoms at admission were fever (100%), splenomegaly and hepatomegaly (100%), pallor of the skin and mucosa (82.5%), edema of any intensity (38.5%), bleeding in one or more sites (25%), and jaundice (7.5%). Petechiae, ecchymosis, hemorrhagic suffusions, gingival bleeding, and enterorrhagia, isolated or associated, were present in 10 (25.64%) patients. 

Laboratory findings at admission showed low levels of hemoglobin 6.82 (1.36) g/dL, leukocytes 3,722 (1,472) /mm^3^, neutrophils 1,161 (671) /mm^3^, platelets 58,384 (30,536) /mm^3^, and prothrombin activity 73.65 (17.81) %. High levels of ferritin 1,497 (1,550) ng/dL, triglycerides 404.71 (230.77) mg/dL, CRP 64.06 (45.73) mg/L, GOT- glutamic-oxaloacetic transaminase 245.58 (380.53) IU/L, GPT- glutamic-pyruvic transaminase 110.33 (177.79) IU/L, total bilirubin 0.75 (0.72) mg/dL, direct bilirubin 0.35 (0.46) mg/dL, and indirect bilirubin 0.39 (0.33) mg/dL were observed. 

Other estimated values were total protein: 6.47 (1.33 ) g/dL; albumin: 2.98 (0.47) g/dL; globulins: 3.59 (1.00) g/dL; creatinine: 0.49 (0.14) mg/dL; urea: 21.81 (7.09) mg/dL; amylase: 42.10 (22.54) IU/L; lipase: 32.23 (25.68) IU/L; potassium: 4.18 (0.53) mEq/L, and sodium: 134.12 (3.83) mEq/L. No electrocardiographic changes were observed on the admission exams. 

The microscopic examination of the bone marrow showed hemophagocytosis in 16/37 (43.24%) patients who were subjected to aspiration puncture. Two patients were not subjected to bone marrow biopsy due to the absence of a hematologist at the time of the procedure. 

Specific tests for the diagnosis of VL were also performed. Among 37 patients, the direct test was positive in 26 (70.3%) patients, negative in 11 (29.72%) patients, and was not performed in two (5.12%) patients. Rapid tests for VL, Kalazar Detect^®^, or IT Leish^®^ were positive in 32/34 (94.11%) patients, negative in 2/34 (5.88%), and not performed in 5/39 (12.82%) patients. 

Antibiotic therapy was used in 31/39 (79.5%) patients, being empirical in 22/39 (56.4%) patients and specific in 9/39 (23.1%); there were 4 patients with pneumonia, 3 with acute otitis media, and 2 with sepsis and disseminated intravascular coagulation (DIC). 

Blood component transfusions were used in 19/39 (48.7%) of patients; 18/39 (46.2%) patients received red blood cell (RBC) transfusions, 7/39 (36.8%) received platelet transfusions, and 3/39 (15.8%) also required fresh frozen plasma. 

Corticotherapy was used in 15/39 patients (38.5%), and dexamethasone, associated with treatment for VL, was prescribed as a therapy for HLH. Anti-inflammatory doses were used in 9/15 patients (60%) and immunosuppressive doses, in 6/15 (40%) patients. Among the 15 patients who used corticosteroids, 6 (40%) were younger than two years old (2 received immunosuppressive doses), and 9 (60%) were older than two years (4 received immunosuppressive doses). The age of the group of patients using corticotherapy ranged from five months to six years and one month.

Dexamethasone at the dose of 10 mg/m² of body surface was used in cases of immunosuppression. Two patients used it for eight weeks, three for six weeks, and one for four weeks. The prescribed anti-inflammatory dose was 0.22 mg.kg^-1^.day^-1^ for seven days.

All patients were negative for the rapid HIV test and were given specific treatment for VL for the first time.

N-methylglucamine antimony administration was the first choice in 18/39 (46.1%) patients, out of which, 10 (25.6%) were cured, and 8 (20.5%) had their treatment interrupted (5 patients (12.8%) due to non-therapeutic response and 3 (7.7%) due to adverse effects. Amphotericin B deoxycholate was prescribed for one patient with a good therapeutic response.

Liposomal Amphotericin B was prescribed as the first option in 20/39 (51.3%) patients: 12/39 (30.8%) patients were administered with 4 mg.kg^-1^.day^-1^ for five consecutive days, and 8/39 (20.5%) patients were administered with 3 mg.kg^-1^.day^-1^ for seven consecutive days. All these patients were cured, but one patient in the 3 mg.kg^-1^.day^-1^ group had his treatment extended for an additional 7 days.

The rescue treatment for VL, with the use of liposomal amphotericin B, was performed in all patients who were not cured. Six patients were administered with 4 mg.kg^-1^.day^-1^ for five consecutive days, and two patients were administered with 3 mg.kg^-1^.day^-1^ for seven days; all 8 of these patients have been cured.

After treatment, all patients were followed up in outpatient clinics for twelve months. Consultations were held in the second, sixth, and twelfth month after treatment or at any time when deemed necessary. The record of follow-ups showed that all the patients were cured at the end of twelve months (Table 1).

**TABLE 1:** Visceral leishmaniasis associated with hemophagocytic lymphohistiocytosis, criteria that defined the diagnosis and therapies performed in thirty-nine pediatric patients with an association of HLH/VL. Period from January 2012 to December 2017.

Nº	Age (years old)	Sex	Hemophagocytosis	Fever days	Hepatosplenomegaly	Cytopenias	Ferritin ng/mL	Triglycerides mg/dL	Jaundice	T. He	T. Plaq	T. PFC	Antibiotic therapy	Corticosteroid therapy	Clinical outcome
1	4a10m	F	POS	13	PRES	Pa	519	401	ABS	R	NR	NR	AE	NR	CURED
2	1a	M	NEG	13	PRES	Pa	≥ 1.500	556	ABS	R	R	R	AI	A	CURED
3	2a1m	M	POS	10	PRES	Pa	≥ 1.500	812	ABS	R	NR	NR	AI	A	CURED
4	11m	M	NEG	7	PRES	Pa	574	580	ABS	R	R	NR	AE	NR	CURED
5	4a11m	M	POS	28	PRES	Bi	≥ 1.500	210	ABS	NR	NR	NR	AE	A	CURED
6	2a3m	M	NR	30	PRES	Pa	≥ 1.500	270	ABS	R	R	NR	AE	A	CURED
7	3a7m	M	NEG	20	PRES	Pa	≥ 1.500	443	ABS	R	NR	NR	AE	S	CURED
8	11m	M	NEG	8	PRES	Pa	512	339	ABS	R	R	NR	AE	A	CURED
9	1a4m	F	NEG	90	PRES	Pa	≥ 1.500	309	ABS	NR	NR	NR	AE	NR	CURED
10	1a3m	F	NEG	15	PRES	Bi	≥ 1.500	359	ABS	R	NR	NR	NR	NR	CURED
11	11m	F	POS	30	PRES	Bi	580	1.620	PRES	NR	NR	NR	AE	S	CURED
12	2a3m	M	NEG	30	PRES	Bi	≥ 1.500	387	ABS	R	NR	NR	AE	A	CURED
13	4a	M	NEG	30	PRES	Bi	≥ 1.500	442	ABS	NR	NR	NR	NR	NR	CURED
14	4a5m	F	POS	15	PRES	Bi	830	286	ABS	NR	NR	NR	NR	NR	CURED
15	4a7m	F	POS	14	PRES	Bi	900	300	ABS	R	R	R	AI	S	CURED
16	1a4m	M	POS	11	PRES	Bi	≥ 1.500	291	ABS	NR	NR	NR	AI	NR	CURED
17	5m	F	POS	15	PRES	Pa	542	280	ABS	R	NR	NR	AE	S	CURED
18	9m	M	NEG	7	PRES	Bi	879	268	ABS	NR	NR	NR	AE	A	CURED
19	4a10m	F	NR	15	PRES	Pa	≥ 1.500	332	ABS	R	NR	NR	AE	A	CURED
20	1a6m	M	POS	15	PRES	Bi	≥ 1.500	467	ABS	R	NR	NR	AI	NR	CURED
21	2a	M	NEG	8	PRES	Bi	≥ 1.500	323	ABS	NR	NR	NR	AE	NR	CURED
22	6a	M	NEG	120	PRES	Pa	1.100	267	ABS	R	NR	NR	AE	NR	CURED
23	3a6m	M	POS	15	PRES	Bi	≥ 1.500	273	ABS	NR	NR	NR	AE	NR	CURED
24	6a1m	F	POS	15	PRES	Bi	≥ 1.500	359	ABS	NR	NR	NR	NR	S	CURED
25	1a3m	F	NEG	60	PRES	Pa	≥ 1.500	391	ABS	NR	NR	NR	AE	NR	CURED
26	1a	F	NEG	20	PRES	Bi	≥ 1.500	301	ABS	NR	NR	NR	AE	NR	CURED
27	9a2m	M	POS	30	PRES	Bi	≥ 1.500	295	ABS	NR	NR	NR	NR	NR	CURED
28	2a6m	M	POS	9	PRES	Pa	≥ 1.500	254	ABS	R	NR	NR	AE	S	CURED
29	1a1m	F	NEG	7	PRES	Bi	1.124	349	ABS	NR	NR	NR	AI	NR	CURED
30	1a10m	F	NEG	20	PRES	Bi	1.297	590	PRES	R	NR	NR	AE	NR	CURED
31	9m	M	NEG	15	PRES	Bi	550	397	ABS	NR	R	NR	AI	NR	CURED
32	4a4m	F	NEG	30	PRES	Bi	≥ 1.500	337	ABS	NR	NR	NR	AE	NR	CURED
33	2a6m	M	NEG	15	PRES	Pa	≥ 1.500	489	ABS	NR	R	NR	AE	NR	CURED
34	1a	M	NEG	15	PRES	Bi	1.461	466	ABS	NR	NR	NR	AE	NR	CURED
35	3a	F	NEG	9	PRES	Bi	≥ 1.500	332	ABS	NR	NR	NR	NR	NR	CURED
36	4a4m	F	POS	21	PRES	Bi	887	381	ABS	NR	NR	NR	NR	NR	CURED
37	1a8m	F	POS	60	PRES	Pa	NR	397	ABS	R	R	NR	AI	A	CURED
38	2a1m	F	POS	20	PRES	Pa	≥ 1.500	263	ABS	NR	NR	NR	NR	NR	CURED
39	8a2m	M	NEG	20	PRES	Pa	673	368	ABS	R	R	NR	AE	NR	CURED

**Source:** Prepared by the author, 2018. **VL:** visceral leishmaniasis; **HLH:** hemophagocytic lymphohistiocytosis; **M:** male; **F:** female; **POS:** positive; **NEG:** negative; **R:** performed; **NR:** not performed; **PRES:** present; **ABS:** absent; **Bi:** bicytopenia; **Pa:** pancytopenia; **T. He, Pla, PFC:** transfusions of packed red blood cells, platelets and fresh frozen plasma; **AE:** empirical antibiotic therapy; **AI:** antibiotic therapy with identified focus; **A:** anti-inflammatory dose; **S:** immunosuppressive dose.

The mean time between the onset of symptoms and admission was 23.72 (22.8) days, with fever reported by all patients as the initial symptom of the disease. The mean hospital stay was 17.74 (5.7) days (ranging from 7 days to 29 days).

## DISCUSSION

Several tropical infections associated with HLH are rapidly increasing. The clinical findings of VL overlap with those of HLH, and using the diagnosis of leishmaniasis as a trigger for the syndrome can be challenging, even in endemic areas. Consequently, the delay in the diagnosis of HLH associated with VL, likely due to clinical similarities, can cause severe complications and death in up to 90% of patients without specific treatment for HLH^10^ . 

HLH associated with VL rarely occurs in childhood, with less than 50 cases reported in the literature until 2012^11^. This finding is corroborated by Bode et al.^12^, who found a 2.1% prevalence of HLH in German patients with VL, mainly in children. Even in India, which accounts for 90% of the cases of VL worldwide, this association has been reported as rare^13^. The same is true for other countries such as Tunisia^14^, Turkey^15^, Sudan^16^, and Iran^17^, with most of the studies being case reports. 

However, the prevalence in Brazil is high. Daher et al. found 35 pediatric patients with HLH among 127 (27.5%) participants with VL in an endemic area in the Northeast region of the country^18^. 

The present study, until the most recent literature review on the subject, has the highest number of reported cases. Conducted in an endemic area in the north of the state of Minas Gerais, it found a prevalence of 15.1% in a series of 258 cases of children aged less than 10 years, who were admitted with a diagnosis of VL. This fact draws attention to the need to consider HLH triggered by VL, especially in patients with an unfavorable course during the specific treatment. However, in countries where VL is not endemic, the presence of secondary HLH is mostly triggered by viral infections, with the Epstein-Barr virus being the most frequent causative agent, with the worst prognosis^19^ ^,^ ^20^. 

In Brazil, pediatric patients have a higher prevalence of the association between VL and HLH compared to adults, which differs from the findings of studies conducted in other countries^4^ ^,^ ^21^ ^-^ ^23^. 

Literature data show that the main trigger for secondary HLH in childhood is viral infections, chiefly the Epstein-Barr virus^19^, which was not observed in this study. During the study period, there was only one case of HLH secondary to this virus, which had an unfavorable course, only reverting with the use of chemotherapy. This patient was not included in this study.

The majority of the diagnosed patients were below two years of age, which is consistent with the findings of other studies^11^ ^,^ ^21^. The mean weight showed no tendency or risk of malnutrition, unlike what has been observed in several previous studies on VL^24^. There was a slight predominance of males over females, which is consistent with the findings of studies by Daher et al. and Scalzone et al^11^ ^,^ ^17^. Moreover, the majority of the diagnosed patients inhabited urban areas, thereby confirming the process of urbanization of VL that has occurred since 1980^25^. 

All patients met five or six laboratory and clinical criteria for HLH and VL, with the maximum number of achievable criteria being six since the institution did not conduct research on NK cell activity or soluble CD25 dosage, which would have allowed for the achievement of eight possible criteria. However, HLH and VL were confirmed by the occurrence of hemophagocytosis in the bone marrow aspirate, along with serum triglycerides and ferritin levels, the latter being measurable up to 1,500 ng/dL. 

Direct parasitological examination of the aspirated bone marrow material and the rapid test for VL (Kalazar detect^®^ or IT-Leish^®^) were the specific tests used. The rapid test showed the best performance, being positive for almost all patients who were evaluated^26^ ^,^ ^27^. It is important to highlight that the last five participants did not undergo the test due to the lack of the product.

The direct examination of bone marrow aspirate showed a similar positive response to that recorded in other studies^28^,^29^ but contrasted with the low positivity found in cases of VL/HLH, as described by Gagnaire et al^21^. The discrepancy in the direct examination is indicative of the relevance of the rapid test, which has high sensitivity and specificity, ease, and speed of execution^26^ ^,^ ^27^, and therefore, is considered useful in patients whose diagnosis and treatment should be initiated as early as possible to avoid the complications that most often cause death.

Fever, hepatosplenomegaly, and pancytopenia were detected in all participants; nearly half presented with involvement of the three peripheral blood lineages, and all had anemia and thrombocytopenia. This high prevalence of clinical and laboratory changes could be explained by the etiopathogenesis of this association, which leads to an intense activation of monocytes and macrophages by cytokines produced by T lymphocytes in response to the infection^28^. It is important to emphasize that the clinical and hemogram similarity between the two entities (fever, hepatosplenomegaly, pancytopenia) often hinders and delays the diagnosis, consequently affecting the treatment^9^ ^,^ ^30^.

The changes described herein are consistent with those described in several other studies on this topic, as reported by Henter et al^9^. 

Complete remission of HLH with VL-specific therapy was observed in most patients. However, a considerable number of patients required the use of corticotherapy in anti-inflammatory (23.0%) or immunosuppressive (15.4%) doses. 

In order to inhibit cytokine expression and suppress the excessive immune response, dexamethasone with a dose of 10 mg/m² of body surface was used as an immunosuppressive agent, as indicated by Gagnaire et al^22^ ^,^ ^31^. The anti-inflammatory dose was 0.22 mg.kg^-1^.day^-1^ for 7 days. This drug was chosen because it can cross the blood-brain barrier, thereby suppressing inflammation in the central nervous system. Another intervention is to use prednisone or prednisolone at the dose of 1 mg.kg^-1^.day^-1^ while the signs of HLH severity persist, especially coagulation disorders^21^. Moreover, Imashuku et al. have recommended using water-soluble corticosteroids (dexamethasone or methylprednisolone or prednisolone) as the first line of treatment^32^. Patients classified as low risk for HLH have shown rapid healing with corticotherapy alone or in association with intravenous immunoglobulin^33^. 

In the present study, all patients were followed up for twelve months after treatment, as suggested by other researchers^32^. The one-year follow-up was scheduled after hospital discharge and was already part of the routine of this service. All patients were cured of VL and its complications, and the use of other therapies was not necessary, as also reported by Henter et al^34^.

Significant improvement in prognosis has been achieved through the incorporation of supportive intensive care at the onset of the disease, including the control of DIC and opportunistic infections associated with neutropenia, and the prompt introduction of immunosuppression to control the cytokine storm^33^. 

Patients in this study were not tested for fibrinogen levels or CD25 and NK cell activity. Despite the limitations, this study contemplates the largest series of cases recorded so far on the association between VL and HLH, which seems to be more frequent than reported by other studies, but with lower mortality.

HLH associated with childhood VL in endemic areas is common and should always be considered since it is a potentially fatal condition.

In the present study, the majority of patients with the VL/HLH association, compared to those with VL alone, were younger than 3 years old and living in urban areas. All had fever, hepatosplenomegaly, anemia, and thrombocytopenia. The clinical manifestations were more exuberant, often showing signs of severity, such as hemorrhage in one or more sites, edema, toxi-infectious state, and jaundice. Additionally, aminotransferases were often elevated. Transfusions of blood products, antibiotics, and corticosteroids were frequently used, in addition to specific treatment for VL.

Early diagnosis and treatment may lead to a cure for HLH. Medical professionals aiding in these areas should be prepared for the diagnostic suspicion when faced with clinical and laboratory conditions that characterize VL (fever, hepatosplenomegaly, and changes in one or more peripheral blood lines) and HLH (Added to the VL signals, elevated serum ferritin levels > 500 µg/L, triglycerides > 265 mg/dL, or fibrinogen <150 mg/dL). Figures of hemophagocytosis in the bone marrow are not exclusive to HLH.

## CONCLUSION

In Brazil, HLH is a common and potentially fatal complication of VL, which pediatricians should be aware of. The diagnosis of HLH should be considered in patients with severe VL, especially if there is evidence of liver involvement. Although the syndrome may be inapparent and healed with anti-parasitic therapy of leishmaniasis, this may not always occur. HLH has been reported to cause high lethality; therefore, immunosuppressive or anti-inflammatory therapy should be instituted immediately to avoid a fatal outcome. Taking into consideration the use of corticotherapy, further studies are needed to indicate the best option. Liposomal Amphotericin B is effective and well-tolerated when used for a short period.
